# Combustible Gas Classification Modeling using Support Vector Machine and Pairing Plot Scheme

**DOI:** 10.3390/s19225018

**Published:** 2019-11-17

**Authors:** Kyu-Won Jang, Jong-Hyeok Choi, Ji-Hoon Jeon, Hyun-Seok Kim

**Affiliations:** Division of Electronics and Electrical Engineering, Dongguk University-Seoul, Seoul 04620, Korea; jgw0911@naver.com (K.-W.J.); yibee1226@naver.com (J.-H.C.); kile156@naver.com (J.-H.J.)

**Keywords:** semiconductor gas sensor, decoupling algorithm, gas classification, pairing plot, support vector machine

## Abstract

Combustible gases, such as CH_4_ and CO, directly or indirectly affect the human body. Thus, leakage detection of combustible gases is essential for various industrial sites and daily life. Many types of gas sensors are used to identify these combustible gases, but since gas sensors generally have low selectivity among gases, coupling issues often arise which adversely affect gas detection accuracy. To solve this problem, we built a decoupling algorithm with different gas sensors using a machine learning algorithm. Commercially available semiconductor sensors were employed to detect CH_4_ and CO, and then support vector machine (SVM) applied as a supervised learning algorithm for gas classification. We also introduced a pairing plot scheme to more effectively classify gas type. The proposed model classified CH_4_ and CO gases 100% correctly at all levels above the minimum concentration the gas sensors could detect. Consequently, SVM with pairing plot is a memory efficient and promising method for more accurate gas classification.

## 1. Introduction

The need for the detection of various gases in industrial and public areas has been continuously increasing as environment, health, and safety issues arise. Combustible gas detection is the most important due to the risk of fire and explosion [1,2,3,4]. Various gas sensor types have been used to detect combustible gases in the atmosphere including electrochemical, semiconductor, photoelectric, and MEMS sensors [5,6]. Semiconductor gas sensors offer many advantages, including low cost, small size, wide range of detectable gases, fast response time, and high sensitivity to combustible gases. However, high and broad sensitivity leads to relatively low selectivity and, consequently, to coupling problems where the sensor reacts to another gas in duplicate or cross-response. Sensor response can be greatly degraded by coupling. Considerable research and development efforts have been focused on physical parameters, such as materials, sensor structure, and sensor driving conditions, etc., to solve this problem, but these approaches have not yet achieved a technical level of commercialization. Therefore, a gas classification algorithm to compensate for the coupling problem may be a more viable solution. Consequently, many studies have considered gas classification models incorporating various machine learning methods [7,8,9,10,11,12,13].

In this study, we constructed a decoupling algorithm with two different SnO_2_ semiconductor gas sensors based on support vector machine (SVM) to classify CH_4_ and CO as representative combustible gases. We also introduced a new pairing plot scheme in the gas classification algorithm to obtain gas detection signal behavior patterns that could be classified into two classes by SVM. An experimental calibrating gas environment was set up and gas sensing experiments were conducted under specific gas injection conditions. After data acquisition, first data selection (FDS) was applied to include only meaningful data in the classification model, and then behavior patterns for each gas were analyzed using pairing plots. Gas sensor responses showed distinguishable patterns. Subsequently, second data selection (SDS) was performed to reduce computational costs. Finally, we built a gas classification model based on non-linear SVM and verified reliability for the final model using a confusion matrix.

## 2. Materials and Methods

**Figure 1:**
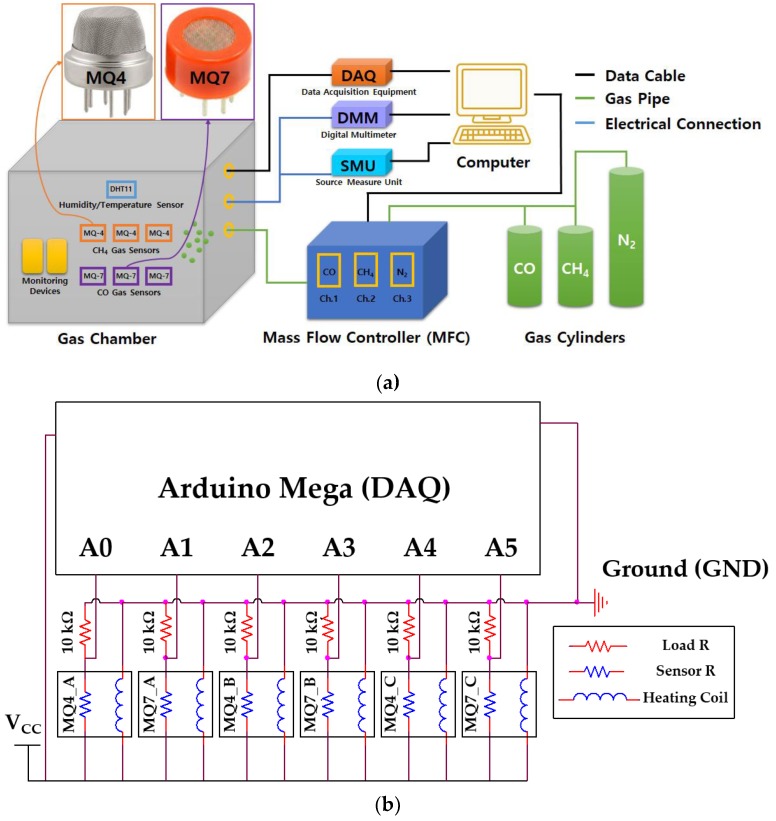
Experiment schematics for gas detection: (**a**) experiment equipment setup including data acquisition equipment (DAQ), digital multimeter (DMM), source measure unit (SMU), and mass flow controller (MFC) and (**b**) gas sensor array and circuit block diagram.

We designed the experimental setup to provide a controllable gas environment, as shown in Figure 1a. The setup included a gas chamber connected with gas cylinders, data acquisition equipment (DAQ) for gas sensor control and measurement, digital multimeter (DMM) to verify electrical signals in the circuit, source measure unit (SMU) for specific voltage supply to the circuit, mass flow controller (MFC) for accurate CH_4_ and CO flow control, and a computer to control these components and run gas classification algorithms.

We employed commercially available MQ4 and MQ7 sensors [14,15] (Zhengzhou Winsen Electronics Technology Corporation, Zhengzhou, China) to detect CH_4_ and CO, respectively, as shown in Figure 1a. These are SnO_2_-based n-type semiconductor sensors [16,17,18,19] which operate based on reactions with combustible gases around the SnO_2_ surface. When the sensor is heated up, oxygen is actively adsorbed on the surface, taking electrons from the SnO_2_ surface, forming an electron depletion region beneath the surface. When CH_4_ and CO gases are present around SnO_2_ with sufficient energy, they react with adsorbed oxygen atoms, subsequently releasing electrons to SnO_2_ and, hence, reducing sensor resistance. Therefore, a load resistor is required in the data collection circuit, and voltage drops across the load resistor increase as the sensor resistance reduces due to the gas interaction. Thus, we collected load resistor voltages as gas detection signals. The load resistance was set 10 kΩ to obtain high sensing resolution which can be determined by following equations:

(1) $$V_{r}=\frac{V_{cc}\times R_{L}}{R_{s\_min}+R_{L}}-\frac{V_{cc}\times R_{L}}{R_{s\_max}+R_{L}}$$

where *V_r_* is the output signal range, *V_CC_* is the operating voltage, *R_L_* is the load resistance, *R_s_max_* is the maximum sensor resistance, and *R_s_min_* is the minimum sensor resistance.

(2) $$V_{r}=\frac{V_{cc}\left(R_{s\_max}-R_{s\_min}\right)}{\frac{R_{s\_max}\times R_{s\_min}}{R_{L}}+\left(R_{s\_max}+R_{s\_min}\right)+R_{L}}$$

Next, maximum *V_r_* in Equation (2) can be obtained by calculating only the minimum value of $\frac{R_{s\_max}\times R_{s\_min}}{R_{L}}+R_{L}$ because *V_CC_, R_s_min_*, and *R_s_max_* are constant.

(3) $$\frac{R_{s\_max}\times R_{s\_min}}{R_{L}}+R_{L}\geq 2\sqrt{\frac{R_{s\_max}\times R_{s\_min}}{R_{L}}\times R_{L}}$$

(4) $$R_{L}=\sqrt{R_{s\_max}\times R_{s\_min}}$$

As shown in Equations (3) and (4), by using arithmetic–geometric mean inequality, the minimum value of $\frac{R_{s\_max}\times R_{s\_min}}{R_{L}}+R_{L}$ is calculated and *R_L_* is decided.

Moreover, the operating temperature also affects the sensors’ performance [20,21,22]. Practically, the best temperatures for CH_4_ and CO to be adsorbed on the SnO_2_ surface are very different, presenting less cross-selectivity with respect to other gases. However, even if sensors operate at the best operating temperature for each gas, the cross-selectivity issue still cannot be fully ignored. This study employed the selectivity differences among the two types of sensor for decoupling. Thus, the operating temperature of each sensor should be constant by fixing an operating voltage of 5 V (*V_cc_*) and the heating coil resistance of each gas sensor. Figure 1b shows that the sensor circuit comprised three cross-arranged MQ4 and MQ7 sensors for effective gas detection.

In this study, we conducted the gas detection experiments for a single gas environment. More specifically, a situation was assumed to identify whether it was CH_4_ or CO when multiple semiconductor gas sensors were employed. Gas injection experiments commenced with aging time to heat and, hence, stabilize the sensors. After sufficient aging time, gas was injected at specific rates (standard cubic centimeter per minute (sccm)) for 20 s. Injection then stopped and a 5 min reaction time was allowed to ensure the gas sensors fully reacted. The same cycle was repeated with increasing gas levels until the target gas concentration was attained. The experiments were carried out under ambient atmosphere, i.e., air for both CH_4_ and CO gas detections since the metal oxide semiconductor sensors are not operational without oxygen. Moreover, N_2_ gas was only employed to purge and remove CH_4_ and CO gases remaining in the gas lines. After conducting each gas experiment, we evacuated CH_4_ or CO gas inside the chamber to initialize the experimental environment. Table 1 shows gas injection conditions for CH_4_ and CO gas detection experiments.

The experimental setup was carried out for each gas concentration within a range that could be fatal to humans by assuming CH_4_ or CO gas was leaked at actual industrial sites or public places. Thus, the target gas concentrations were different, because the human hazardous concentration of CH_4_ gas is higher than the CO gas according to the dangerous concentration criteria of the Korea Gas Safety Corporation and Korea Environment Corporation.

We designed the SVM for classifying CH_4_ and CO gases using MATLAB^®^. In general, SVM is a machine learning method classifying two or more data classes [23,24,25,26,27]. This study built a classification model with non-linear SVM to classify curved behavioral patterns. In the SVM algorithm, kernel function helps modeling for non-linear hyperplane with reduced computational costs. Thus, we employed a Gaussian RBF kernel function, which is one of the generally used and high-performance functions, which can be expressed as;

(5) $$k\left(X_{i},Y_{i}\right)=exp\left(-\gamma \|X_{i}-Y_{i}{\|}^{2}\right)$$

where *X_i_* and *Y_i_* are data set vectors corresponding to CH_4_ and CO gas, respectively; and *γ* is a parameter controlling the deviation of the Gaussian function [28,29]. After gas classification modeling, we verified the classification model’s reliability using a confusion matrix with test data sets extracted from a distinct gas detection experiment [30,31,32]. A confusion matrix is a visualization method for classification of model performance and reliability. The model’s reliability verification using the confusion matrix proceeded with new data sets that did not belong to the training data. The confusion matrix visualizes the matches between the predicted class and the true class. We also used several dummy data sets to double check classification model reliability.

## 3. Results

**Figure 2:**
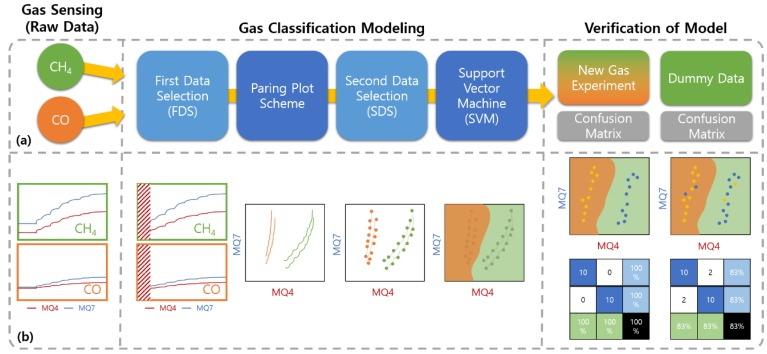
Overall conceptual diagram for gas classification: (**a**) the classification flow for the entire processes and (**b**) the output schematics for each process.

As shown in Figure 2, the overall procedure of the gas classification consisted of gas sensing experiments, gas classification modeling, and two-step verifications. Moreover, gas classification modeling involves four steps: first data selection (FDS), pairing plot scheme, second data selection (SDS), and SVM.

### 3.1. Raw Data for Gas Sensing

**Figure 3:**
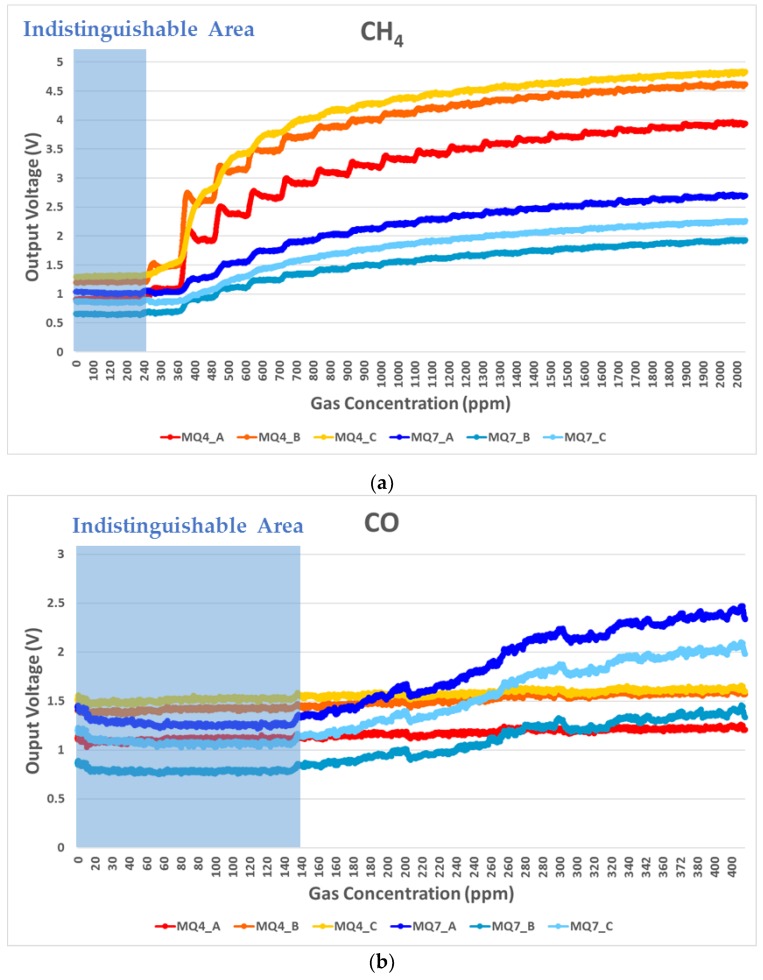
Measured representative sensor output voltages for gas detection experiments: (**a**) CH_4_ and (**b**) CO gas.

Figure 3 shows output voltages for the load resistor resulting from MQ4 and MQ7 sensor reactions with respect to gas concentration. These output voltages were logged every 2 s by DAQ. Therefore, we could confirm the responses of each gas sensor by observing the voltage changes from the load resistor.

Although the MQ4 sensor was specific for detecting CH_4_ gas, it also reacted to CO gas with a similar issue arising for the MQ7 sensor. Thus, both sensors exhibit low selectivity and, hence, coupling problems for gas signals. Therefore, it was not possible to clearly identify CH_4_ or CO gas levels from either sensor alone. Even using both gas sensors, it was difficult to classify gas type from output voltages alone. Therefore, we proposed SVM with a pairing plot method.

### 3.2. Pairing Plot Scheme for Support Vector Machine

There were concentration ranges where the sensing signals were indistinguishable (Figure 3, blue marked area) due to the gas sensors’ physical limitations. Thus, we needed to select meaningful data before pairing the data, i.e., FDS. It was necessary to avoid confusion about the initial response of the sensors due to the noise voltages under ambient atmosphere. To extract meaningful data used for machine learning, the SnO_2_ gas sensor signals should be distinguishable from the initial detection value (*V_initial_*) at which sensors start detecting gases. The noise voltage difference (*V_noise.diff_*) is the difference between maximum and minimum noise voltage values before gas injection. *V_initial_* should be at least two times larger than *V_noise.diff_*. Based on these criteria, we specified indistinguishable sensing value ranges as shown in Figure 3 (blue marked area). We set the FDS criteria based on the *V_initial_* for each gas sensor, defined by ambient atmosphere voltage (*V_ambient_*) and *V_noise.diff_*. In short, the *V_initial_* was determined by the minimum detectable voltage (*V_det_*) in the following equations:

(6) $$V_{det}\geq V_{ambient}+\left(V_{noise.diff}\times 2\right)$$

(7) $$V_{det.min}=V_{initial}$$

where *V_ambient_* is average output voltage in ambient atmosphere, and *V_noise.diff_* is the difference between maximum and minimum noise voltage in ambient atmosphere.

**Figure 4:**
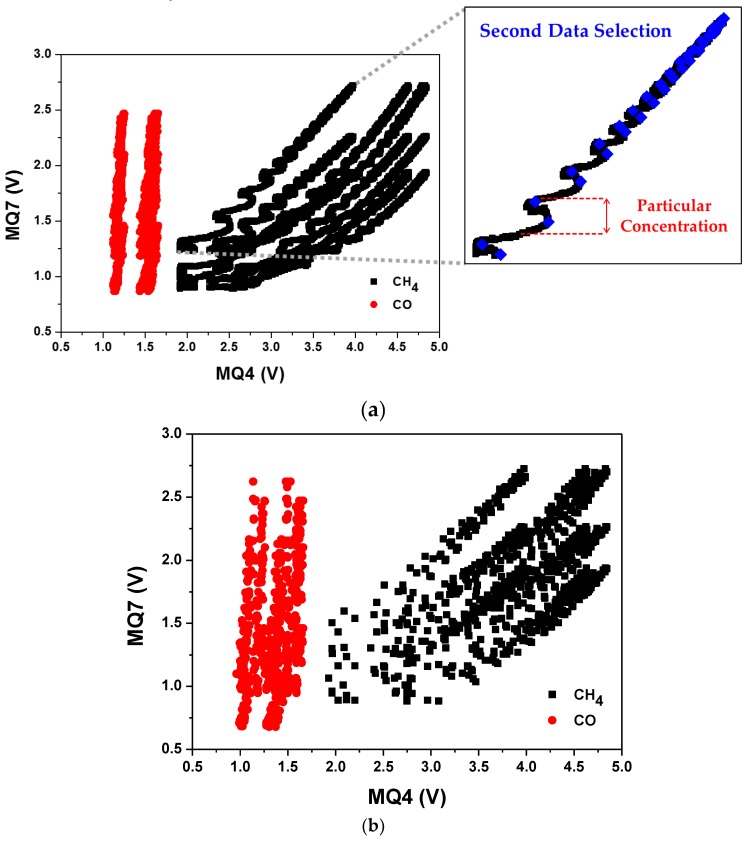
Pairing plots for the CH_4_ and CO gas detection data sets: (**a**) all possible pairing cases with first data selection for one experiment data (inset: second data selection detail) and (**b**) second data selection for all experiment data sets.

Only output voltages above *V_initial_* were selected for the pairing plot. The selected data were plotted in the form of (MQ4, MQ7) considering all possible pairing cases in each gas detection experiment. For example, since there were three MQ4 and three MQ7 sensors, nine pairing (MQ4, MQ7) cases were extracted from each experiment. Figure 4a shows the pairing plots for the FDS applied gas detection experiment. The CH_4_ and CO gas had distinguishable behavior patterns that enabled them to be clearly classified.

In SVM training, determining hyperplane was performed using only boundary data for each class. Thus, for the data sets selected from FDS, additional data selection was performed using the concentration in each injection cycle. This provided significant memory and computational efficiency for the learning process. Figure 4a inset shows that the number of data in the particular gas concentration can be reduced to two data points through SDS by pairing the maximum MQ4 value with the corresponding MQ7 value and the maximum MQ7 value with the corresponding MQ4 value, i.e., (MQ4_max, MQ7) and (MQ4, MQ7_max), respectively, providing the pairing plot with the minimum number of essential data (Figure 4b). We subsequently applied non-linear SVM with these paired data sets.

### 3.3. Gas Classification Model Using Non-Linear Support Vector Machine

**Figure 5:**
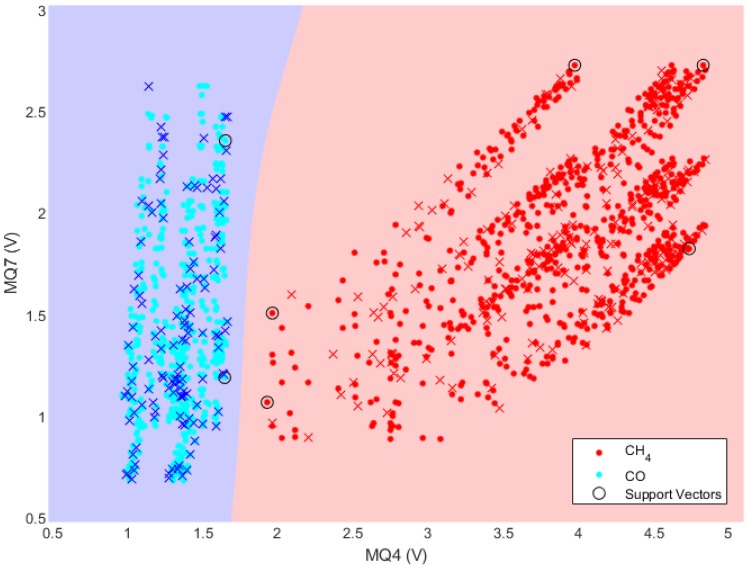
CH_4_ and CO gas classification using non-linear SVM with pairing plots; • = training and **×** = testing data sets.

Selected data sets were randomly divided into training and testing data sets at a 4:1 ratio. Feature selection for the data sets was decided by the output voltage, since all data sets only included the output voltage in this study. The K-Fold cross validation was used to avoid the overfitting problem for the training data sets [33,34,35]. The most important SVM step is to find the hyperparameters defining the optimal hyperplane. We used the Gaussian RBF kernel method for the non-linear SVM to define the hyperplane and, hence, establish the classification model. Subsequent verification with the testing data sets confirmed 100% classification accuracy. Figure 5 shows the visualization of elements for the defined hyperplane, support vectors, and all data sets.

**Figure 6:**
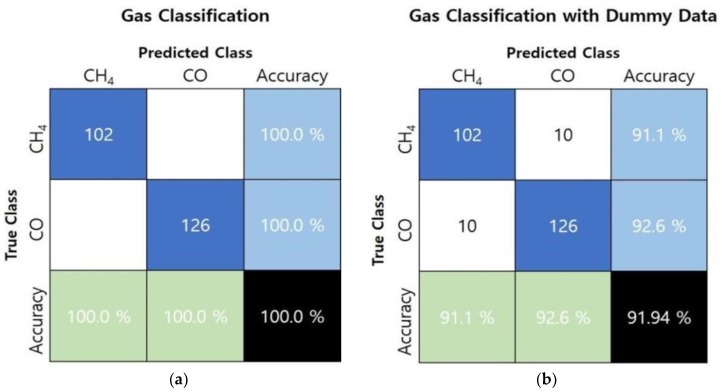
Confusion matrix for classification model with (**a**) data sets from new gas detection experiments and (**b**) an additional 20 incorrect data sets.

To verify classification model reliability, we extracted paired data sets for a new CH_4_ and CO gas detection experiment. The number of data sets for CH_4_ and CO were 102 and 126, respectively. Figure 6a shows the gas classification confusion matrix for the new paired data sets, confirming 100% classification accuracy for each gas. Moreover, we intentionally created 10 paired data sets with incorrect values for each gas to double check the model’s reliability. As shown in Figure 6b, the confusion matrix results for classification by adding these dummy data sets were visualized. Consequently, the reliability of the non-linear SVM gas classification model was verified again by fully classifying all 20 incorrect paired data sets.

## 4. Conclusions

Although combustible gas detection in industrial and public areas is essential, it is difficult to accurately identify gases due to the inferior semiconductor gas sensor performance. In particular, selectivity issues cause significant coupling problems among sensing signals, making accurate gas identification difficult. Thus, it is necessary to introduce an algorithmic approach to compensate for this issue. In this work, we proposed a classification algorithm based on support vector machine by introducing a pairing plot technique. Furthermore, we achieved the memory efficient gas classification model using the data selection method. Model reliability was verified by classifying CH_4_ and CO gases 100% accuracy through additional tests with confusion matrix. Thus, the proposed method classified CH_4_ and CO gases simultaneously with 100% accuracy even in the presence of gas sensor selectivity issues. The proposed approach is not specific to semiconductor gas sensors and could also be applied to most or all other sensor types which have sensing signal coupling problems. Therefore, modeling with non-linear support vector machine and pairing plot technique could be an effective way to identify gases.

## Figures and Tables

**Table 1:** CH_4_ and CO gas injection conditions for gas detection experiments.

Parameter	Unit	CH_4_	CO
Gas injection rate	sccm	30	6
Gas injection time	sec	20	20
Reaction time	min	5	5
Gas injection concentration	ppm	100	20
Total number of gas injections	-	20	20
Target gas concentration	ppm	2000	400
